# Current knowledge and perspectives of potential impacts of *Salmonella enterica* on the profile of the gut microbiota

**DOI:** 10.1186/s12866-020-02008-x

**Published:** 2020-11-17

**Authors:** Nesreen H. Aljahdali, Yasser M. Sanad, Jing Han, Steven L. Foley

**Affiliations:** 1 Division of Microbiology, National Center for Toxicological Research, U.S. Food and Drug Administration, 3900 NCTR Rd, Jefferson, AR 72079 USA; 2 Biological Science Department, College of Science, King Abdul-Aziz University, Jeddah, Saudi Arabia; 3 Department of Agriculture, University of Arkansas, Pine Bluff, AR USA; 4 Department of Parasitology and Animal Diseases, Veterinary Research Division, National Research Centre, Giza, Egypt

**Keywords:** Gut microbiota, *Salmonella enterica*, Host cell-micbobe interaction

## Abstract

In the past decade, the initial studies of the gut microbiota started focusing on the correlation of the composition of the gut microbiota and the health or diseases of the host, and there are extensive literature reviews pertaining to this theme. However, little is known about the association between the microbiota, the host, and pathogenic bacteria, such as *Salmonella enterica*, which is among the most important foodborne pathogens and identified as the source of multiple outbreaks linked to contaminated foods causing salmonellosis. Secretion systems, flagella, fimbriae, endotoxins, and exotoxins are factors that play the most important roles in the successful infection of the host cell by *Salmonella*. Infections with *S. enterica*, which is a threat to human health, can alter the genomic, taxonomic, and functional traits of the gut microbiota. The purpose of this review is to outline the state of knowledge on the impacts of *S. enterica* on the intestinal microbiota and highlight the need to identify the gut bacteria that could contribute to salmonellosis.

**Supplementary information:**

**Supplementary information** accompanies this paper at 10.1186/s12866-020-02008-x.

## Background

An enteric pathogen is a microbe that impacts the gastrointestinal tract (GIT) and causes gastrointestinal diseases. These infectious pathogens, including bacteria such as *Escherichia*, *Campylobacter*, *Shigella*, *Yersinia*, *Salmonella*, and other genera, protozoa such as amoeba, rotavirus, and other pathogenic microorganisms, are responsible for causing gastroenteritis [1]. Among enteric pathogens there is often an age-associated bias with the development of gastroenteritis upon exposure. For example, *Escherichia coli* (*E. coli*) causes enteric disease in people most commonly during early and late ages, whereas rotaviruses are the most common among infants and young children. Similarly, *Campylobacter* infections occur most often in early childhood into young adulthood, while *Salmonella* infections have higher rates in infants and people over 65 [2]. *Salmonella* infections are a significant global public health threat and contribute to morbidity and mortality worldwide [3]. The *Salmonella* genus is generally considered to be divided into two species: *S. enterica* and *S. bongori*. Although, *S. bongori* appears adapted to cold-blooded animals, it can infect humans, but accounts for less than 1% of human infections [4, 5]. On the other hand, several of the subspecies of *S. enterica* are more commonly isolated from warm-blooded animals. *S. enterica* includes six subspecies: *S. enterica* subsp. *enterica*, *S. enterica* subsp*. salamae*, *S. enterica* subsp. *arizonae*, *S. enterica* subsp. *diarizonae*, *S. enterica* subsp. *houtenae*, and *S. enterica* subsp. *indica*. Among these subspecies, *S. enterica* subsp*. salamae*, and *S. enterica* subsp. *arizonae*, are more commonly isolated from cold-blooded animals [6, 7]. *S. enterica* includes more than 2600 serotypes that differ from each other based on the polysaccharide portion of lipopolysaccharide layer (O antigen) and/or the filamentous portion of the flagella (H antigen) [8]. Scallan et al (2011) estimated that nontyphoidal *Salmonella* account for approximately 28% of foodborne illness-associated deaths [9]. The predominant subspecies associated with severe disease is *S. enterica* subsp. *enterica* and among its serotypes, there is also variability in the outcomes of disease with some serovars causing relatively severe outcomes. For example, *S. enterica* serovar Heidelberg contributes to about 7% of the *Salmonella*-related deaths in the U.S. [10] and 11% of reported invasive infections, which are relatively high percentages considering that they typically cause under 5% of infections [11].

*S. enterica* is a highly diverse Gram-negative bacterial species that can be divided into typhoidal and nontyphoidal *Salmonella* serovars. Typhoidal *Salmonella* serovars share virulence properties that were obtained through convergent evolution and therefore these virulence genes are absent from most  non-typhoidal *Salmonella* serovars [12]. For instance, *S*. Typhi has specific virulence factors, including typhoid toxin and Vi antigen [7, 12, 13]. Nontyphoidal *S.* including Typhimurium, Enteritidis, Heidelberg, Newport, Weltevreden, Choleraesuis, Saintpaul, Infantis and Javiana cause gastroenteritis, while typhoidal *S.* including Typhi and Paratyphi serovars commonly cause typhoid fever [13]. Nontyphoidal serotypes can be transferred between humans and animals, whereas typhoidal serotypes are only transmissible among humans [14]. Notably, nontyphoidal *Salmonella* disseminates rapidly in people with an impaired immune system and in neonates [15]. Ninety-five percent of *S. enterica* infections are associated with consumption of contaminated food products [7]. More than 2600 serotypes of *Salmonella* have been identified [16, 17]. To clarify links between *Salmonella* serotypes and food products, Jackson and colleagues (2013) indicated that more than 80% of outbreaks caused by serotypes Enteritidis, Heidelberg, and Hadar were associated with eggs or poultry, while greater than 50% of outbreaks caused by serotypes Javiana, Litchfield, Mbandaka, Muenchen, Poona, and Senftenberg were attributed to plant commodities. Serotypes Typhimurium and Newport were linked to a wide variety of food commodities [18]. These organisms invade the GIT causing salmonellosis, which is typically characterized by a self-limiting gastroenteritis symptom, such as diarrhea, fever, abdominal cramps, and vomiting [19].

The GIT is host to diverse taxa from across the tree of life, such as bacteria, archaea, fungi, protozoa, and viruses that make up the gut microbiota [20]. The gut harbors a highly diverse microbial community, which impacts the host’s nutrition, physiology, and immune system [21, 22]. The composition of the gut microbiota remains relatively stable within healthy people throughout their lifetime [23]. However, specific shifts in the composition and diversity have been linked to diet, diseases, and susceptibility to infection. For instance, alteration of the intestinal microbiota has been associated with acute inflammation that can be triggered by enteric pathogens [24]. *Salmonella* and other pathogens have been widely studied; however, the interactions between enteric pathogens and intestinal microbes are not well understood. In this review we will summarize the knowledge of the interaction between *Salmonella* and intestinal microbiota that is currently available and clarify the research that needs to be undertaken to understand the consequences of theses interactions.

### Gut microbiota

#### Human gut microbiota/microbiome

**Fig. 1:**
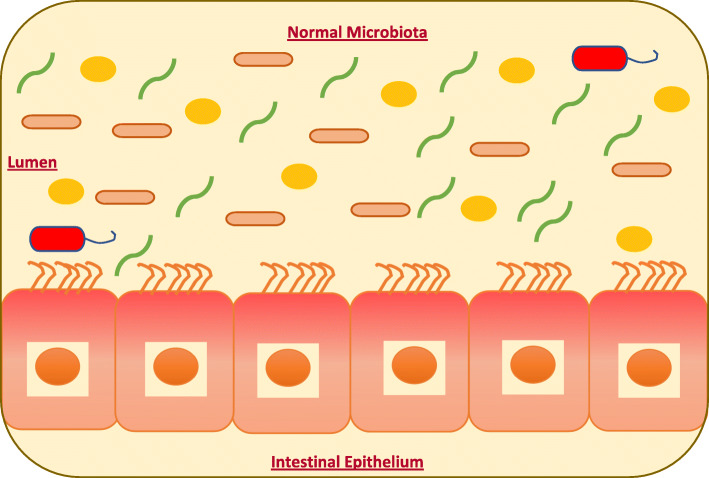
Normal gastrointestinal tract of humans harbors the high relative abundance of commensal bacteria, such as Bacteroidetes and Firmicutes with smaller portions of Actinobacteria, Verrucomicrobia, and Proteobacteria

The human body hosts up to 100 trillion (10^14^) microbes, with the majority residing in the GIT, which has become the most investigated microbial community in recent years [20, 25]. Most of the microbiota in the GIT are primarily anaerobic bacteria. Typically, 97% of the bacteria in the GIT are strict anaerobes, and only 3% constitute the aerobic bacteria (facultative anaerobes) [26]. The collective pan-genome of bacterial cells is larger than the human genome [25]. There are large differences in microbial load in different regions of the GIT. To illustrate this, *Helicobacter pylori* resides in the stomach at a concentration of 10^2^–10^3^ cells/ml. The mucosa of the small intestine is dominated by the phyla Bacteroidetes and members of the *Clostridiales* cluster XIV and IV*,* and the lumen contains members of the *Enterobacteriaceae* with a biomass of 10^4^–10^5^ cell/ml [22, 25]. The large intestine contains species from the phyla Bacteroidetes and Firmicutes with amounts in the range of 10^11^–10^12^, with other phyla including Proteobacteria, Verrucomicrobia, and Actinobacteria being less represented (Fig. 1) [22, 25].

Generally, the composition of the gut microbiota shifts throughout life as people transition from newborns to infants to young adults to elders. The GIT of newborns is expected to be sterile at birth. However, major shifts take place during and immediately after birth due to the colonization with aerobic bacteria (*Enterococcaceae* and *Streptococcus*) [27]. The gut microbiota composition of infants is highly dynamic with low levels of total bacteria [28]. The microbiota of infants is dominated by some members of *Clostridium, Bifidobacteria,* and facultative anaerobes like *E. coli*, while elderly people generally have higher levels of *Bacteroidetes* and facultative anaerobes like *E. coli* [29]. In young adults the composition of the gut microbiota is dominated by Bacteroidetes and Firmicutes with smaller portions of Actinobacteria, Verrucomicrobia, and Proteobacteria [25].

A generally symbiotic relationship between the host and the gut microbiota has been known to be strongly associated with health [22]. The host provides a nutrient-rich and hospitable environment for the gut microbiota. In parallel, the gut microbiota is extremely important as it supports the host by enhancing metabolism, maturation of the immune system, developing the GIT, and protecting against pathogens [26, 30]. Also, intestinal bacteria degrade undigested foods by two main metabolic pathways: saccharolytic and proteolytic pathways. Non-digestible carbohydrates are degraded into monomeric sugars that can be converted to beneficial products, such as short-chain fatty acids (SCFAs), principally acetate, propionate, and butyrate. These products have been shown to decrease the risk of developing gastrointestinal disorders, cancer, and other metabolic syndromes [31–33]. Peptide and amino acids, on the other hand, are hydrolyzed into short or branched-chain fatty acids and other metabolic elements, some of which are possibly toxic to the host, such as uremic toxins [34, 35]. The gut microbiota usually lives within the host in a commensal manner; however, many external factors can alter the balance of this microbiota composition, potentially leading to gastrointestinal diseases, such as salmonellosis.

### Salmonellosis

#### *Salmonella* gastroenteritis

*Salmonella* infections are significant economic and public health concerns, costing an estimated 3.7 billion dollars per year [36, 37]. According to the Centers for Diseases Control and Prevention (CDC), it is estimated that members of the *Salmonella* genus cause 1.35 million infections leading to 26,500 hospitalizations and 420 deaths per year in the United States [38]. Salmonellosis can manifest in several disease syndromes including *Salmonella* gastroenteritis, inflammation, enteric fever, bacterium, and other syndromes [39, 40]. *Salmonella* gastroenteritis is the predominant form of salmonellosis and is characterized by stomach cramps, diarrhea, fever, and sometimes vomiting [3]. Human salmonellosis is most commonly associated with consumption of contaminated foods, resulting in the ability of *Salmonella* to colonize and persist in the GIT [7, 41–43]. It has been reported that the highest hospitalization rates are among the elderly and young children [38, 44]. As previously mentioned, the gut microbiota composition of infants and old people are highly dynamic with higher percentages of facultative anaerobes like *E. coli* [29]. Thus, the ability of *Salmonella* to invade the GIT is relatively high when the bacterial population of the GIT is less stable due to higher levels of Proteobacteria [2]. Furthermore, young children have immune systems that are still developing (immunocompromised) that also contributed to their higher prevalence of salmonellosis compared to adults [45]. Details of the interactions of *Salmonella* and the GIT will be explored in greater detail throughout the review.

The plasticity of bacterial genomes is known in *Salmonella* species to influence the acquisition of genes through horizontal and vertical gene transfer [46]. This plasticity can be achieved with the presence of mobile genetic elements (MGEs), such as plasmids [11]. Plasmids play vital roles in the ability of *Salmonella* to survive in different food animal sources and cause infections in humans [9]. Plasmids are self-replicating genetic elements that can allow for gene transfer between different bacteria. The presence of plasmids can impact the ability of *S. enterica* to cause disease and avoid treatment strategies due to the presence of antimicrobial resistance and virulence genes that they carry. These factors have allowed for the dissemination of epidemic clones over large geographical distances that have contributed to significant morbidity and mortality [47, 48]. Several plasmid types have been identified carrying antimicrobial resistance and virulence genes [7]. Horizontal gene transfer, with plasmids or other MGEs, can impact the host range of the bacterium [7]. The acquisition of genes can be important for colonization of pathogens in the host cell. *S. enterica* and other pathogens can enter the host’s GIT through the fecal-oral route, and the effector proteins they harbor can manipulate and overcome the intestinal epithelial barrier [49]. The ability of *Salmonella* and other enteric pathogens to invade the GIT is relatively high when the colonic microbiota is less stable due to higher numbers of Proteobacteria during infections [2]. Despite the role of the intestinal epithelium as a protective barrier against bacterial infections, the genetics of *Salmonella* itself play a significant role in survival and growth in diversified host environments [7, 50]. Several strategies allow *S. enterica* to effectively compete with the gut microbiota and overcome colonization, such as the expression of an assortment of virulence factors and the exploitation of intestinal inflammatory processes.

*S. enterica* harbor the *Salmonella* pathogenicity island-1 (SPI-1) encoded type III secretion system (T3SS) and *Salmonella* pathogenicity island-2 (SPI-2) encoded T3SS, which facilitate the attachment, invasion, and internalization of *Salmonella* during infection in the host cell. To illustrate, *S*. Typhimurium contains genes, such as those for the *Salmonella* invasive proteins (Sips) and *Salmonella* outer proteins (Sops) encoded in the SPI-1 T3SS. These proteins alter the actin cytoskeleton of intestinal epithelial cells, resulting in membrane ruffling and bacterial internalization [51]. Furthermore, SopE induces nitrate production by the host, which boosts *Salmonella* growth in the host cell [52]. Once *Salmonella* is engulfed within intestinal epithelial cells, the host cell membrane is rearranged leading to the formation of a membrane-bound organelle termed a *Salmonella* containing vacuole (SCV), where *Salmonella* can replicate to high numbers before exiting the cell and infecting new host cells [53]. The SPI-2 T3SS genes are expressed inside the SCV, resulting in the rapid induction of intestinal inflammation [54]. In addition to SPIs, plasmids, carrying virulence genes, are essential for the infection process to host cells in order to ensure nutrient supply [55], compete against commensal bacteria [56], avoid killing by innate immune system, and manipulate the host to establish infection [57].

#### Inflammatory response

The innate immune system plays a crucial role in controlling infections when *Salmonella* has been detected. To illustrate, the O-antigen and lipid A of *Salmonella* are detected by the innate immune system elements including complement component 3 and macrophages, which result in the production of pro-inflammatory cytokines, such as IL-22, IL-18, TNF-α, and other cytokines [58]. Thereby, the induction of cytokines culminates the host defense pathway, including neutrophil recruitment, macrophage activation, and the release of an antimicrobial protein [24]. Cattle infected with *S.* Typhimurium displayed a massive infiltration of neutrophils following infection [59]. Neutrophils limit pathogen loads in the mucosa and in the intestinal lumen at later stages of infection [60]. Macrophages also contribute to pathogen clearance; for instance, proteins called toll-like receptors (TLRs) on the surface of macrophages can recognize pathogen-associated molecular patterns (PAMPs) and eliminate the pathogens [61]. Moreover, macrophages produce nitric oxide (NO), which diffuses across cellular membranes to combat pathogens [62]. Additionally, during *S.* Typhimurium infection, IL-18 plays a vital for induction of inflammation within the first 12 h of infection and recruits neutrophil and mature natural killer (NK) cells to the site of infection. The NK cells express perforin, which plays a major role in the induction of mucosal inflammation [63]. This inflammation plays important roles in the pathogenesis of *Salmonella* in the GIT.

Microbial communities play a fundamental role in regulating immunity in the GIT [22]. The intestinal microbiota mediates colonization resistance against enteric pathogens through activation of antimicrobial host immune mechanisms. For instance*, Lactobacillus reuteri* plays an important role in the induction of IL-22, a cytokine that enhances the mucosal barrier against pathogens [58, 64]. Another important support of the immune response modulated by the microbiota involves the stimulation of IL-1B, which results in the recruitment of neutrophils to the site of the infection [65]. However, infections with *Salmonella* result from competition with the gut microbiota during an intestinal inflammatory response [66]. To illustrate this phenomenon, during a *S.* Typhimurium infection, neutrophils that migrate into the lumen of the colon release reactive oxygen species (ROS), which oxidizes thiosulfate to form tetrathionate that can be used by *S*. Typhimurium as an anaerobic respiratory electron acceptor allowing for competition with the microbiota [24, 67]. Moreover, NO, which is produced by macrophage, can be exploited by *Salmonella* and used to generate nitrate, which can be used as a terminal electron acceptor [52].

**Fig. 2:**
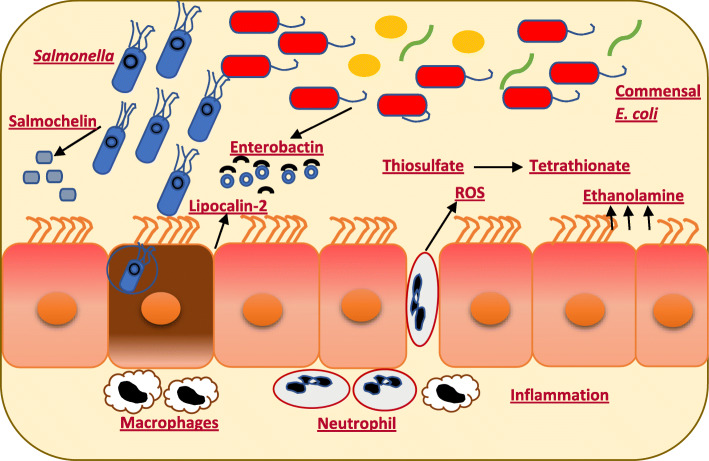
During infection with *Salmonella*, the gut shifts to the low relative abundance of commensal bacteria such as, *Lachnospiraceae*, *Clostridiales* with a higher portion of members of *Enterobacteriaceae*, *E. coli****.*** Neutrophils migrate and release ROS, which oxidizes thiosulfate to tetrathionate used by *Salmonella*. Lipocalin-2 release from the intestinal lumen and bind to enterobactin, but not salmochelin

The more rapid growth of *S.* Typhimurium in the intestine is due in part to its ability to utilize ethanolamine, which is released from the epithelial tissue [68]. After inflammation is induced, lipocalin-2, a host antimicrobial protein is released into the intestinal lumen in response to IL17- and IL-22 [69]. Lipocalin-2 binds to enterobactin that is produced by members of the *Enterobacteriaceae* in the microbiome, but not salmochelin that is produced by *Salmonella* [70]. The sequestration of enterobactin, but not salmochelin, allows for the *S*. Typhimurium to bloom in the lumen of the inflamed intestine and result in a bacteriostatic activity for some commensal bacteria, such as *E. coli* [70, 71]. Additionally, *S.* Typhimurium induces expression of colicin Ib and Ia genes, which increase the fitness of *S.* Typhimurium in competition against commensal *E. coli* [21]. Thus, *Salmonella* elicits an acute intestinal inflammatory response from the host, which enhances its transmission and growth in the GIT. Once the *Salmonella* has colonized the GIT, the alteration of the gut microbiota composition and the horizontal gene transfer (HGT) between *Salmonella* and commensal bacteria can occur (Fig. 2).

### Interaction between gut microbes and *Salmonella*

#### Alteration microbial composition in the gut caused by *S. enterica*

The number of *Enterobacteriaceae* is relatively low when the gut microbiota has developmentally stabilized in the GIT [25]. The microbiol communities produce a diversity of products, such as SCFAs, secondary bile acids, and bacteriocins that provide resistance against colonization by pathogens in the GIT. The commensal microbiota protects the host from enteric pathogens [72]. For example, in an in vivo study, microcin, produced by *E. coli* Nissle (EcN), can limit the growth of competing *Enterobacteriaceae*, including commensal *E. coli*, and pathogenic *Salmonella* in the inflamed gut [73]. Conversely, infections with *Salmonella* can impact the host intestinal microbial composition (Table 1). A recent study found that infections with *S.* Typhimurium resulted in the alteration of the gut microbiota composition in the ceca of pigs. There were significant increases in the population of *Anaerobacter, Barnesiella, Pediococcus, Sporacetigenium, Turicibacter, Catenibacterium, Prevotella, Pseudobutyrivibrio,* and *Xylanibacter* in the infected pigs compared to the control groups [74]. Furthermore, in an in vivo setting, *S.* Typhimurium infections in pigs impacted the microbial diversity at the ileum mucous. This change was reflected in a rise in numbers of the potentially pathogenic bacteria *Citrobacter,* with a corresponding decrease in *Bifidobacterium, Lactobacillus*, and *Ruminococcus*, which are often considered beneficial to gut health [75]. Moreover, it was reported that infections with *S.* Typhimurium resulted in a reduction of specific microbiota species, such as SCFA-producing bacteria [76]. More recently it was found that *S.* Typhimurium-infected mice disturbed the gut microbiota composition with an increase in the relative abundance of *Enterobacteriaceae*, including *Enterobacter cancerogenus*, *Proteus penneri*, and *Escherichia fergusonii*, but an overall decrease in bacterial diversity [77]. Barman et al (2008) found that infections with *S.* Typhimurium resulted in the reduction of the total bacterial number in the cecum and large intestine of mice [78]. They found the relative abundances of *Lactobacillus, Enterococcus, Eubacterium rectale*, and *Clostridium coccoides* were significantly lower in *S.* Typhimurium infected mice compared to uninfected controls [78].

**Table 1:** Summary of the effect of *S. enterica* on the gut microbiota composition

*S. enterica*	Impact of infection on gut microbiota	Method for Analyses of Gut Microbiota	Reference
*S.* Typhimurium infected pig	Increase in *Anaerobacter, Barnesiella, Pediococcus, Sporacetigenium, Turicibacter, Catenibacterium, Prevotella, Pseudobutyrivibrio,* and *Xylanibacter*	Roche 454 GS-FLX sequencer	[74]
*S.* Typhimurium infected pig	Increase *Citrobacter* but decrease *Bifidobacterium, Lactobacillus*, *Clostridium spp*., and *Ruminococcus*	Illumina MiSeq sequencer	[75]
*S.* Typhimurium-infected mice	Increase *Enterobacteriaceae* members, such as *Enterobacter cancerogenus*, *Proteus penneri*, and *Escherichia fergusonii*	Illumina MiSeq sequencer	[77]
*S.* Typhimurium-infected mice	Decrease *Lactobacillus spp., Enterococcus spp., Eubacterium rectale*, and *Clostridium coccoides*	Quantitative real-time PCR amplification	[78]
*S.* Enteritidis infected chicken	Increase *Anaerotruncus, Bacillus, Enterococcus, Anaerostipes, Flavonifractor* and *Intestinimonas* but decrease *Blautia, Shuttleworthia*, and *Anaerostipes*	Illumina MiSeq sequencer	[79]
*S.* Enteritidis infected young chicken	Increase *Enterobacteriaceae* members but decrease *Lachnospiraceae* family	Illumina MiSeq sequencer	[80]
*S.* Enteritidis infected chicken	Increase *Enterobacteriaceae* family but decrease *Ruminococcaceae members*	Pyrosequencing 454 sequencer	[81]
*S.* Enteritidis infected chicken	Increase *Enterobacteriales* bacteria but decrease *Clostridiales*, *Lactobacillales*, and *Bifidobacteriales*	Quantitative real-time PCR amplification	[82]

**Fig. 3:**
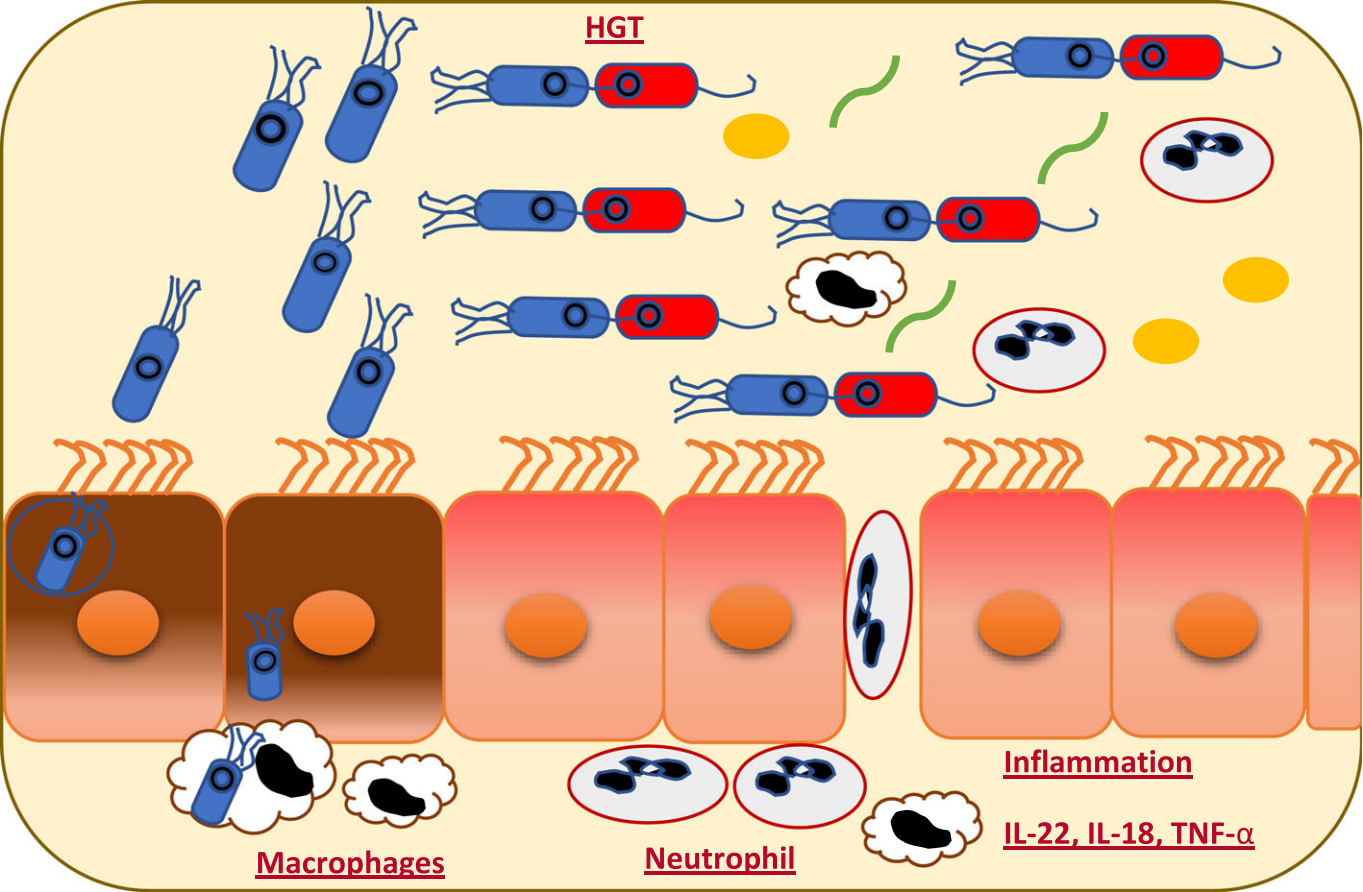
During infection with *Salmonella*, the horizontal gene transfer (HGT) can occur between *Salmonella* and commensal bacteria, such as commensal *E. coli*

Similar findings demonstrated that *S.* Enteritidis can affect the composition of the gut microbiota by changing the relative abundance of certain microbes. It was found that chickens inoculated with *S.* Enteritidis over an extended period had an altered relative abundance of genera at different time points [79]. *Blautia, Shuttleworthia*, and *Anaerostipes* were less abundant, but *Anaerotruncus, Bacillus, Enterococcus, Anaerostipes, Flavonifractor* and *Intestinimonas* were more abundant in the infected chicken than the control group [79]. Another study found that the relative abundance and the overall diversity of the microbiota populations significantly changed at the family level after infections with *S.* Enteritidis [80]. The study demonstrated that *Salmonella* colonization in the GIT of the chicken had a significant inverse correlation between the *Enterobacteriaceae* and *Lachnospiraceae* families, with an increase of *Enterobacteriaceae* members [80]. Also, a previous report studying hatched chicks found that infection with *S*. Enteritidis caused a minor numerical increase in the members of *Enterobacteriaceae*, but *Ruminococcaceae* decreased, although these results were not significant [81]. Likewise, Juricova et al (2013) demonstrated that infections with *S.* Enteritidis can alter the number of bacteria at the order taxonomic level [82]. The relative abundance of *Enterobacteriales* was higher in the infected chickens than in the control group. This increase corresponded to a decline in the relative abundance of *Clostridiales*, *Lactobacillales*, and *Bifidobacteriales* [82] (Fig. 3). Interestingly, it is important to note that there are other pathogens that can impact the diversity and abundance of the gut microbiota. Thus, there is interest to know how other pathogenic bacteria can alter the composition of the gut bacteria. Previous studies have indicated that the intestinal communities in patients with enteric bacterial infections had lower species richness and diversity, compared to apparently healthy people [83]. For instance, patients infected with different pathogens, such as *Campylobacter*, *Salmonella*, *Shiga* toxin-producing *E.coli* (STEC), and *Shigella* had high abundance of Proteobacteria members, while higher abundances of Bacteroidetes and Firmicutes were observed in healthy people [83]. The study found that the relative percentage of Proteobacteria was different between the populations colonized with different pathogenic bacteria. To illustrate, the relative abundances of Proteobacteria was 37% in patients infected with *Campylobacter*, followed by 29% with *Salmonella*, 18% with STEC, and 38% with *Shigella* [83]. Furthermore, the authors noted that genus *Escherichia* predominated in the fecal microbiome of patients infected with pathogens such as *Campylobacter, Salmonella*, *Shigella* and STEC, where the mean percentage of *Escherichia* were 0.21, 0.14, 0.24, and 0.21, respectively, compared to uninfected people (0.01) [83]. Thus, once the alteration of the microbial profile in the GIT happens, the effective conjugative transfer can occur among bacteria [21].

#### Horizontal gene transfer between *S. enterica* and commensal bacteria

HGT or lateral gene transfer (LGT) is the exchange of genetic material between unicellular and/or multicellular organisms by means other than by the vertical transmission of genetics between generations [84]. A few recent studies have started to focus on the prevalence of antimicrobial resistance (AMR) genes in the commensal microbiota. The gut microbiota shows greater rate of HGT than that of bacteria in other environments [85]. HGT can occur via three main mechanisms: transformation, transduction or conjugation [86]. Persistent temperature, nutrient influx, and the high relative abundance of microbes in the gut form an appropriate environment for HGT among bacteria. The plasticity of microbial metagenome is believed to be attributable to HGT between microbes [87, 88]. It has been reported that different bacteria can carry identical genes [89]. For example, a study reported that a bile salt hydrolase (*bsh*) gene, encoding resistance to bile found in *Bacteroides, Bifidobacterium, Clostridium, Lactobacillus*, and *Enterococcus*, could be obtained by HGT [90]. The members of *Enterobacteriaceae* are prime examples by which conjugation-mediated HGT has occurred at a relatively high rate in the inflamed gut [21]. In normal gut, the proportion of *Enterobacteriaceae* is very low compared to other taxa. Thus, effective conjugative plasmid transfer is low among the *Enterobacteriaceae* due to the low density of donor and recipient bacteria causing a decreased rate of conjugation-mediated HGT [21, 91].

Although, contact-dependent conjugation between *Enterobacteriaceae* is inhibited by commensal microbiota, the inflammatory response to pathogens can boost the frequency of conjugative HGT [21]. Infections with enteric *Salmonella* can cause *Enterobacteriaceae* to thrive, which can lead to increased HGT between *S. enterica* and commensal microbes (Table 2). Consequently, the intestinal microbiota can act as reservoir for virulence and antimicrobial resistance genes [87, 92]. Stecher and colleagues (2012) found that the colicin-plasmid p2 was able to transfer from *S.* Typhimurium to commensal *E. coli* at a high rate in an in vivo mouse colitis model [21]. Another study found that the transfer of a p3464b plasmid, which carried *bla*_*CTX-M-9*_ resistance gene, from *S.* Virchow isolated from a chicken farm to *E. coli* happened at a higher rate in vivo than in in vitro studies [93]. Further, Faure et al (2010) confirmed that this resistance plasmid was transferred from *S.* Virchow to a commensal *E. coli* isolated from the human GIT using a gnotobiotic mouse model [94]. A recent study demonstrated that pIFM3844 plasmid, harboring multidrug resistance genes and *bla*_*CTX-M1*_ gene, was transferred from *S.* Typhimurium to commensal *E. coli* in an in vitro chicken gut model at a relatively high rate [95]. In early study, Aviv et al (2016) found that pESI megaplasmid, carrying multidrug resistance and virulence genes, can be horizontally transferred to commensal *E. coli* of the mice gut microbiota from *S*. Infantis [96].

**Table 2:** Summary of horizontal gene transfer between *S*. *enterica* and commensal bacteria

Mobile genetic elements	*S. enterica*	Reference
Colicin-plasmid p2	From *S.* Typhimurium to commensal *E. coli.*	[21]
p3464b plasmid carrying *bla*_*CTX-M-9*_ gene.	From *S.* Virchow isolated from a chicken farm to *E. coli.*	[93]
Plasmid carrying *bla*_*CTX-M-9*_ gene.	From *S.* Virchow originating from poultry to a commensal *E. coli* isolated from human.	[94]
pIFM3844 plasmid carrying multidrug resistance genes and *bla*_*CTX-M1*_ gene.	From *S*. Typhimurium to commensal *E. coli*.	[95]
pESI megaplasmid carrying multidrug resistance and virulence genes.	From *S*. Infantis to commensal *E. coli*	[96]
pSA831R plasmid carrying *bla*_*TEM-3*_ gene.	From members of the family *Enterobacteriaceae* to *S.* Anatum in the GIT of patients.	[97]
plPl849 plasmid carrying *bla*_*TEM-3*_ gene.	From *Klebsiella pneumoniae* to *S.* Kedougou in the GIT of individual patients.	[98]
72-MDa plasmid carrying *bla*_*cmy-2*_ gene.	From *E. coli* to *S.* Newport isolated from turkey.	[99]
IncK2-plasmid carrying *bla*_*CMY-2*_ gene.	From *E. coli* to *S*. Heidelberg isolated from chicken.	[100]
R plasmid encoded resistance to streptomycin.	From *E. coli* to *S. Lomita* in the GIT of sheep.	[101]

On the other hand, plasmid-mediated antibiotic resistance transfer may also occur in the opposite direction, from the commensal bacteria to *S. enterica*. For example, a study suggested that pSA831R plasmid carrying the *bla*_CTX-M-3_ gene, encoding resistance to ceftriaxone found in *S.* Anatum, could be acquired from other members of the family *Enterobacteriaceae* through the exchange of genetic materials in the GIT of patients [97]. Archambaud et al (1991) found that *S.* Kedougou isolated from the stools and a blood culture of a patient likely acquired a plPl849 plasmid carrying *bla*_TEM*-*3_ gene from *Klebsiella pneumoniae* in the GIT of individual patients [98]. Also, the 72-MDa plasmid containing *bla*_CMY-2_ gene was likely transferred from *E. coli* to *S.* Newport present in the GIT of turkeys [99]. Another study found that *S.* Heidelberg acquired an IncK2 plasmid carrying *bla*_*CMY-2*_ gene from commensal *E. coli* after inoculation of *S*. Heidelberg into chicken ceca in an in vitro study [100]. Smith (1977) found that R plasmid encoded resistance to streptomycin could be transferred from *E. coli* to *S. Lomita* in the GIT of sheep [101]. Plasmids and other mobile genetics elements not only can be transmitted between *S. enterica* and commensal bacteria, but also can be transferred among diverse bacteria to disseminate genes into a variety of interacting bacterial communities. It would be very interesting to know another horizontal gene transfer can occur among microorganisms.

#### HGT among other microorganisms associated with the GIT

Genes can be disseminated among microorganism in both in vitro and in vivo studies (Table 1**supplement**). It was shown that resistance plasmids that contain genes encoding resistance to at least 14 antibiotics were transferred from *Serratia liquefaciens* isolated from the urine of a patient to *E. coli* originating from humans [102]. Likewise, the transfer of plasmids carrying multiple antimicrobial resistance genes from *K. pneumoniae* isolated from patient to the *E. coli* K12 strain occurred at a relatively high rate in the GIT of mice, compared to an in vitro assay [103]. Another study found that IncI1 plasmid carrying an extended-spectrum β-lactamase gene was able to be transferred from *E. coli* originating from poultry to *E. coli* isolated from a human [104]. Interestingly, plasmids can be conjugatively transferred from Gram-negative to Gram-positive bacteria in some cases [105]. Trieucuot et al (1987) demonstrated that the pAT187 plasmid encoded resistance to kanamycin (*aphA-3*) could be transferred from *E. coli* to *Enterococcus faecalis, Streptococcus lactis, Streptococcus agalactiae, Bacillus thuringiensis, Listeria monocytogenes and Staphylococcus aureus* [105]. On the other hand, the conjugal transfer of the plasmid could also occur from Gram-positive to Gram-negative bacteria. To illustrate, in an in vitro assay it was found that the pBR322-pAMII1 chimeric plasmid designated pATl91, encoding resistance to kanamycin (*aphA-3*), erythromycin (*erm*), and β-lactamase, could be transferred from *E. faecalis* to *E. coli* [106]. Likewise, in germ-free mice, the pBR322-pAMβ1 chimeric vector designated pAt191 plasmid, encoding resistance to kanamycin (*aphA-3*), was transferred from *E. faecalis* to *E. coli*, indicating that the conjugation could account for the resistance gene flux in bacteria observed in the GIT [107]. Shoemaker and colleagues (2000) confirmed that the Gram-negative *Bacteroides* species were able to acquire *erm(B)* and *tet(Q)* genes, encoding resistance to erythromycin and tetracycline from *E. faecalis* and other Gram-positive bacteria in the GIT of patients [108]*.* Because the GIT contains densely populated bacteria, there is opportunity for the transfer of genetic elements among bacteria in the GIT. The cumulative set of antimicrobial resistance genes that is harbored by the gastrointestinal microbiota is called the gastrointestinal resistome [109, 110]. Therefore, there is considerable interest to understand as to what extent bacteria can disseminate these genes in the GIT [111].

Further evidence for conjugative transfer of resistance genes carried by transposons is illustrated by the members of Firmicutes in the GIT. It was shown that transposon Tn*1545*, which carries multiple drug resistance determinants such as those for kanamycin (*aphA-3*), erythromycin (*ermAM*), and tetracycline (*tetM*), can be transferred from *E. faecalis* to *L. monocytogenes* in the GIT of gnotobiotic mice at a high rate, compared to in vitro experiments [112]. Moubareck and colleagues (2003) found that transposon Tn*1546*, which carries *vanA* and multiple other antibiotic resistance genes, such as *ermB*, *tet(L)*, *ant (6)*, and *tetM*, can be horizontally transferred from *E. faecium* originating from pigs to *E. faecium* isolated from humans at a high frequency in the GIT of gnotobiotic mice [113]. This study suggested that different resistance genes can be conjugatively transferred from an *E. faecium* strain of animal origin to a human-origin bacterium of the same species [113]. Earlier studies found that the transposon Tn*1546 * carrying *vanA* gene was transferred from an *E. faecium* isolate of chicken origin to an *E. faecium* isolate of human origin in the intestines of human volunteers [114]. Likewise, another study confirmed that the *vanA* gene, encoding resistance to vancomycin, can be transferred from *E. faecium* originating from pigs and poultry to *E. faecalis* originating from human in the GIT of gnotobiotic mice [115]. Launay and colleagues (2006) demonstrated that transposon Tn*1549*, which carries the *vanB2* gene, can be transferred from *Clostridium symbiosum* to *E. faecium* and *E. faecalis* in the GIT of gnotobiotic mice at a high rate, compared to in vitro experiments [116]. Also, another study confirmed that the *vanB* gene, encoding resistance to vancomycin, was transferred among *E. faecium* in the GIT of patients [117]. It is of central importance to know that conjugative transfer of genes can occur among Bacteroidetes members in the GIT. For instance, it was found conjugative plasmid (pRRI4), encoding to tetracycline resistance gene, was transferred from *Prevotella ruminicola* to *Bacteroides spp* [118]. A study indicated that the transfer of Tn*5030* carrying clindamycin resistance (*ermFU*) gene can occur among *Bacteroides* species [119]. Conjugal transfer of plasmids and conjugative transposons among bacteria appears to be important to the HGT in the GIT. Consequently, the efficiency of HGT among bacteria can be affected by several factors such as SOS response, stress hormones, antibiotic treatment, inflammation, and bacteria-derived factors such as quorum sensing molecules. It is of considerable interest to know the factors that influence HGT.

#### Factors influencing HGT with *S. enterica*

The findings from recent studies indicated that the antibiotic-induced SOS response, which is a global stress response to DNA damage, could promote HGT in bacteria. For example, Bearson and Brunelle (2015) found that the induction of SOS response by antibiotics, such as fluoroquinolones (ciprofloxacin, enrofloxacin and danofloxacin), could facilitate the transfer of plasmid from *S.* Typhimurium DT120 and DT104 to a recipient kanamycin-susceptible *Salmonella* [120]. Furthermore, there is also increasing evidence that the impact of antibiotic intake on increased HGT is 3-fold stronger in the resistome of people treated with antimicrobials compared to untreated people (19 and 5%, respectively) [121]. In addition to antibiotics, norepinephrine (NE), a stress hormone, can contribute significantly to HGT between bacteria. A recent study found that NE enhanced HGT of a conjugative plasmid carrying AMR genes from *S.* Typhimurium to commensal *E. coli* due to upregulated expression of *tra* genes in the presence of NE [122]. A major factor that can influence HGT is inflammation in the GIT. Stecher et al (2012) found that infections with *S.* Typhimurium resulted in an inflammatory response, which prompted HGT of the colicin-plasmid p2 from *S*. Typhimurium to commensal *E. coli* [21].

Moreover, other studies reported that some gut bacteria-derived factors associated with quorum sensing may promote HGT [123]. Quorum sensing signaling molecules are synthesized by gut microbiota and function to control population density and synchronize bacterial behaviors [124]. One important class of signaling molecules are referred as autoinducers, which are the major signaling molecules involved in quorum sensing. The concentration of autoinducers increase as the bacteria replicate and increase in number allowing for sensing of population densities [125]. The most common class of autoinducers are acyl homoserine lactones (AHLs) [124]. *S. enterica* and other Gram-negative bacteria encode SdiA, which is a homolog of the well characterized AHL sensor LuxR, but they do not synthesize their own AHLs [126]. However, *S.* Typhimurium use SdiA as a sensor to detect and respond a variety of AHLs in GIT [127], and potentially influence HGT in the GIT [123]. Interestingly, MuCuddin et al (2006) found that the rumen protozoa are a influencing factor in bacterial gene transfer, enhancing transfer a plasmid carrying the *bla*_CMY-2_ gene from *Klebsiella* to *Salmonella* in both in vitro and in vivo studies of bovine, caprine, and ovine species [128].

#### Promotion or inhibition of *S. enterica* growth by gut bacteria

**Fig. 4:**
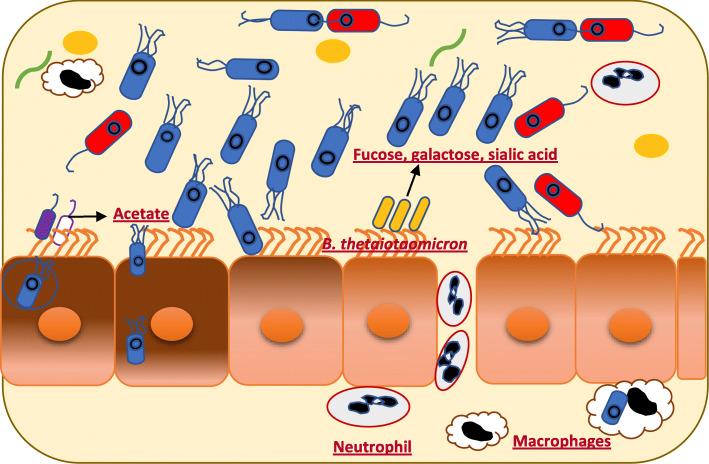
*Bacteroides thetaiotaomicron*, harvests the fucose, galactose, sialic acid from the gut epithelium and used as a source of carbon by *Salmonella* to promote its expansion in the GIT. Acetate produced by commensal microbiota enhanced expression of the invasion genes of SPI-1 encoded T3SS of *Salmonella*

*Salmonella* infections lead to changes in the gut microbiota composition, certain gut bacteria harvest molecules that serve as nutrients or signals to aid in promotion or limitation of the growth of *Salmonella* [129, 130]. The exploitation of microbiota-derived molecules is a critical issue for both the colonization or decolonization of the host cells by enteric pathogens (Table 3). For example, *Bacteroides thetaiotaomicron* harvests the fucose, galactose, sialic acid from the gut epithelium [129, 131, 132]. These sugars can be used as a source of carbon by *S.* Typhimurium to promote its expansion in the GIT (Fig. 4) [129]. Also, hydrogen, which is a central intermediate of microbiota metabolism, can be used as an energy source to enhance the growth of *S.* Typhimurium during the early stages of infection [133]. This growth was enhanced by *S*. Typhimurium *hyb* hydrogenase, which facilitates consumption of hydrogen [133]. Moreover, SCFAs that are produced by members of the GIT microbiota play an important role in colonization of pathogenic bacteria in the GIT. To illustrate, it was shown that the high concentration of acetate in the distal ileum enhanced the expression of the invasion genes of SPI-1 encoded T3SS through sensor kinase (BarA) and response regulator (SirA) pathways (Fig. 4) [134]. In brief, acetate can be converted to acetylphosphate by acetate kinase (AckA), which could phosphorylate BarA and SirA. SirA is essential for the expression of SPI-1 invasion genes [134].

**Table 3:** Summary of certain members of the gut microbiota promotion or inhibition of *S. enterica* growth in the GIT

Gut microbiota	Type of molecules produced by gut microbiota	The result of study	Reference
*Bacteroides thetaiotaomicron*	Fucose, galactose, sialic acid	Enhance the growth of *S.* Typhimurium	[129, 131, 132]
Microbiota-derived H2	Hydrogen (H2)	Enhance the growth of *S.* Typhimurium during the early stage infection	[133]
Microbiota- derived SCFAs	Acetate	Enhance the expression of the invasion genes of SPI-1 encoded T3SS of *S.* Typhimurium	[134]
Microbiota- derived SCFAs	Propionate and butyrate	Suppress the expression of the invasion genes of SPI-1 encoded T3SS of *S.* Typhimurium	[134]
Microbiota- derived SCFAs	Propionate	Limit *S.* Typhimurium growth	[130]
Microbiota- derived SCFAs	Propionate and butyrate	Decrease the invasion of the intestinal epithelial cells in an in vitro avian model of *S*. Enteritidis	[135]
*Lactobacillus casei*	Linoleic acids	Limit *S.* Typhimurium growth	[137]
*Commensal E. coli*	Indole	Downregulated genes of SPI-1 encoded T3SS of *S.* Typhimurium	[139, 140]

Conversely, propionate and butyrate suppressed the expression of the invasion genes of SPI-1 encoded T3SS [134]. Jacobson and colleagues (2018) demonstrated that the production of propionate by *Bacteroides spp*. limited the growth of *S.* Typhimurium by disrupting intracellular pH homeostasis in an in vivo study [130]. Another study found that pre-incubation of *S*. Enteritidis with propionate and butyrate could decrease the invasion of the intestinal epithelial cells in an in vitro avian model [135]. Although the intestinal microbiota is complex and the role of most of the bacteria in providing benefit to the host is not clear, bacterial species of the genera *Lactobacillus* have been shown to supply protection against enteric infections. Peng and colleagues (2015) suggested that *Lactobacillus casei* could inhibit the growth of pathogens by 99% [136]. More specifically, they found that linoleic acids that were produced by *L. casei*, limited the growth of *S.* Typhimurium [137]. Furthermore, Makras et al (2006) found that the inhibitory activity of four of six examined *Lactobacillus* strains against *S*. Typhimurium was solely due to lactic acid production, while that of the remaining two was due to lactic acid plus another unknown substance [138]. In addition, indole is produced by commensal *E. coli* and could be important in the intestinal epithelial cell response to pathogens [139]. Evidence provided by the authors observed that indole downregulated the expression of the SPI-1 T3SS genes of *S*. Typhimurium [140]. Remarkably, there are other pathogens that can also exploit nutrients or molecules for the successful infection of host cells, and it would be very interesting to know other pathogenic bacteria compete and use the molecules harvested by the gut bacteria (Table 2**supplement**).

It has been found that fucose harvested by *B. thetaiotaomicron* repressed the expression of virulence genes in enterohaemorrhagic *E. coli* (EHEC) serovar O157:H7 encoded T3SS through the FusK and FusR signaling cascade [132]. On the other hand, *B. thetaiotaomicron* modified the metabolites by increasing succinate, which can lead to enhance EHEC virulence gene expression through the transcription factor, Cra, which is functionally sensitive to succinate [141]. Takao et al (2014) found that butyrate produced by the gut microbiota enhanced the expression of *leuO* gene that activated the locus for enterocyte effacement (LEE) genes and flagella biosynthesis genes in EHEC-encoded T3SS [142]. Likewise, butyrate enhanced the expression of the Shiga toxin (Stx) receptor globotriaosylceramide (Gb3) on the colonic epithelium and increased susceptibility to EHEC infection [143]. Acyl-homoserine lactones (AHLs) produced by some members of Bacteroidetes can be used by EHEC through sensor protein SdiA to successfully colonize in the intestinal epithelium of cattle [144]. However, molecules that are modified by the gut microbiota can be detected by pathogens and control their virulence genes. For instance, the metabolic conversion of bile acids into deoxycholic acid by some members of the gut bacteria, such as *Bifidobacterium bifidum*, can decrease the expression of virulence genes in *Vibrio cholerae* encoded type VI secretion system (T6SS), which is used to kill other bacteria [145].

## Conclusion

The human gastrointestinal microbiota is a complex of microorganisms that has received much attention because of its impact on human health and disease. Recent insights into the interaction between *Salmonella*, the host and its microbiota, found that *Salmonella* has evolved molecular machineries that allows them to adapt to the inflamed intestine and compete with the gut microbiota. Thereby genes can be transferred horizontally between pathogens and microbial communities that lead to changes in the GIT bacterial structure and their behavior. Together, this interplay could result in risks to human health, for example, the human colon can serve as an environment that acts as a reservoir for antimicrobial resistance and mobile genetics elements. The transfer of MGEs harboring multiple resistance genes and virulence factors from pathogens to human intestinal bacteria has centered around the questions such as: what happens to the transferred MGEs once entering the gut microbiota, and which mechanisms that certain gut bacteria use for HGT can contribute to increasing the virulence factors associated with salmonellosis? Although several recent studies started to focus on understanding the shifts in the taxonomic composition of the developing microbiota from infancy to adulthood; the review of the literature showed that much remains to be learned due to the limited knowledge of the effect of *Salmonella* infection on the microbial composition, as well as on the MGEs in the gut microbiota, including the transmission and persistence of antimicrobial resistance genes.

*S*. Typhimurium has been widely studied as a pathogen and is known to create its own niche in the intestine by causing inflammation, alteration the composition of gut microbiota, and using nutrients produced by gut microbiota. We reviewed many of the latest insights describing the interactions between the microbiota, the host, and pathogenic bacteria in animal models, and it is evident that further studies are needed to better understand the interaction of the gastrointestinal microbiota of different hosts and *Salmonella* serotypes most associated with infections. *S*. Typhimurium has developed mechanisms to rapidly transfer the genes into the gut bacteria at a higher rate in vivo than found in in vitro studies. Although the gut microbiota likely influences *S*. Typhimurium infection kinetics, the effects of molecules produced by gut bacteria on the expression of virulence genes in *S*. Typhimurium is not yet well defined. SCFA produced by bacteria may have utility as therapeutics targets or the for successful prevention against *Salmonella* infection. Likewise, approaches to impact quorum sensing pathways in *Salmonella* and other enteric pathogens could potentially minimize the role of HGT on AMR and virulence factor transmission conserving potential therapeutic options for control of infections.

## Supplementary information

**Additional file 1.**

## Data Availability

Not applicable.

## Abbreviations

GIT: Gastrointestinal tract
SCFAs: Short-chain fatty acids
MGEs: Mobile genetic elements
SPI-1: *Salmonella* pathogenicity island-1
SPI-2: *Salmonella* pathogenicity island − 2
T3SS: Type III secretion system
Sips: *Salmonella* invasive proteins
Sops: *Salmonella* outer proteins
SCV: *Salmonella* containing vacuole
NK: Natural Killer
TLRs: Toll-like receptors
PAMPs: Pathogen-associated molecular patterns
NO: Nitric oxide
ROS: Reactive oxygen species
HGT: Horizontal gene transfer
EcN: *E. coli* Nissle
STEC: *Shiga* toxin-producing *E. coli*
LGT: Lateral gene transfer
AMR: Antimicrobial resistance
MLS: Macrolides, lincosamide and streptogramin
NE: Norepinephrine
BarA: Sensor kinase
SirA: Response regulator
AckA: Acetate kinase
EHEC: Enterohaemorrhagic *E. coli*
AHLs: Acyl-homoserine lactones
T6SS: Type VI secretion system
