# Supplementary material for: Current knowledge and perspectives of potential impacts of Salmonella enterica on the profile of the gut microbiota
Source: BMC Microbiol. 2020 Nov 17;20:353. doi: 10.1186/s12866-020-02008-x (PMC7673091; doi:10.1186/s12866-020-02008-x)
Supplement: Supplementary file 1 — Additional file 1. [file 12866_2020_2008_MOESM1_ESM.docx]

**Table 1 supplement: Example of horizontal gene transfer among microorganisms:**

| **Mobile genetic elements** | **Microorganisms** | **Reference** |
| --- | --- | --- |
| R-plasmid carrying multiple resistance genes. | From *Serratia liquefaciens* isolated from the urine of a patient to *E. coli* originating from human. | [103] |
| Plasmid carrying multiple antimicrobial resistance genes | From *Klebsiella pneumoniae* isolated from patient to *E. coli* K12 strain*.* | [104] |
| IncI1 plasmid carrying extended-spectrum β-lactamase gene. | From *E. coli* to originating from poultry to From *E. coli* isolated from human. | [105] |
| pAT187-plasmid encoded resistance to kanamycin(*aphA-3*). | From *E. coli* to *E. faecalis*, *S. lactis*, *S. agalactiae*, *B. thuringiensis*, *L. monocytogenes* and *S. aureus* | [106] |
| pBR322-pAMII1 chimeric plasmid designated pATl91plasmid encoded to kanamycin (*aphA-3*), erythromycin (*erm*), and β-lactamase genes. | From *E. faecalis* to *E. coli.* | [107] |
| pBR322-pAMβ1 chimeric vector designated pAt191 plasmid encoded to resistance to kanamycin(*aphA-3*). | From *E. faecalis* to *E. coli.* | [108] |
| Transposon carrying *erm(B)* and *tet(Q)* genes. | From *E. faecalis* and other Gram-positive to Bacteroides species. | [109] |
| Transposon *Tn1545* carrying (*aphA-3*), (*ermAM*), and (*tetM*) genes. | Form *E. faecalis* to *Listeria monocytogenes.* | [113] |
| Transposon Tn*1546* carrying *vanA, ermB*, *tet(L)*, *ant(6)*, and *tet(M)* genes. | From *E. faecium* isolate of porcine to *E. faecium* human*.* | [114] |
| Transposon Tn*1546* carrying *vanA* genes. | From an *E. faecium* isolate of chicken origin to an *E. faecium* isolate of human. | [115] |
| Transposon Tn*1549* carrying *vanB2* gene. | From *Clostridium symbiosum* to *E. faecium* and *E. faecalis.* | [117] |
| Plasmid (pRRI4) carrying to tetracycline resistance gene. | From *Prevotella ruminicola* to *Bacteroides spp* | [119] |

**Table 2 supplement: Example of pathogenic bacteria use the molecules harvested by the gut bacteria:**

| **Gut microbiota** | **Type of molecules produced by gut microbiota** | **The result of study** | **Reference** |
| --- | --- | --- | --- |
| *Bacteroides thetaiotaomicron* | Fucose | Suppress the expression of virulence genes in Enterohaemorrhagic *E. coli* (EHEC) encoded T3SS | [133] |
| *Bacteroides thetaiotaomicron* | Succinate | Enhance the expression of virulence genes in EHEC encoded T3SS | [142] |
| Microbiota- derived SCFAs | Butyrate | Enhance the expression of *LeuO* gene that activated *LEE* gene and flagella biosynthesis genes in EHEC encoded T3SS | [143] |
| Microbiota- derived SCFAs | Butyrate | Enhance the expression of Stx receptor Gb3 and then increase the EHEC infection | [144] |
| Some members of Bacteroidetes | Acyl-homoserine lactones (AHLs) | Enhance the colonization of EHEC in the intestinal epithelium | [145] |
| *Bifidobacterium bifidum* | Deoxycholic acid | Decrease the expression of virulence genes in *Vibrio cholerae* encoded T6SS | [146] |
